# Noninvasive metabolic profiling for painless diagnosis of human diseases and disorders

**DOI:** 10.4155/fsoa-2015-0014

**Published:** 2016-06-10

**Authors:** Mainak Mal

**Affiliations:** 1 Freelance science writer, 40 T.C. Road, Flat #2A, Kolkata 700053, India

**Keywords:** biomarkers, exhaled breath, feces, metabolic profiling, metabolomics, noninvasive diagnosis, saliva, urine

## Abstract

Metabolic profiling provides a powerful diagnostic tool complementary to genomics and proteomics. The pain, discomfort and probable iatrogenic injury associated with invasive or minimally invasive diagnostic methods, render them unsuitable in terms of patient compliance and participation. Metabolic profiling of biomatrices like urine, breath, saliva, sweat and feces, which can be collected in a painless manner, could be used for noninvasive diagnosis. This review article covers the noninvasive metabolic profiling studies that have exhibited diagnostic potential for diseases and disorders. Their potential applications are evident in different forms of cancer, metabolic disorders, infectious diseases, neurodegenerative disorders, rheumatic diseases and pulmonary diseases. Large scale clinical validation of such diagnostic methods is necessary in future.

## Abstract (lay abstract)

**Lay abstract:** Metabolic profiling involves determination of changes in metabolite levels caused by diseases and can help in their diagnosis. The pain, discomfort and chance of injury associated with painful diagnostic methods, make them unsuitable in terms of patient compliance. Metabolic profiling of body fluids like urine, breath, saliva, sweat and feces, which can be collected in a painless manner, could be used for painless diagnosis. This review article is about such studies, which have exhibited painless diagnostic potential for diseases and disorders. Their applications are evident in cancer, metabolic disorders, infections, neurodegenerative disorders, rheumatic diseases and lung diseases.

**Figure 1.**
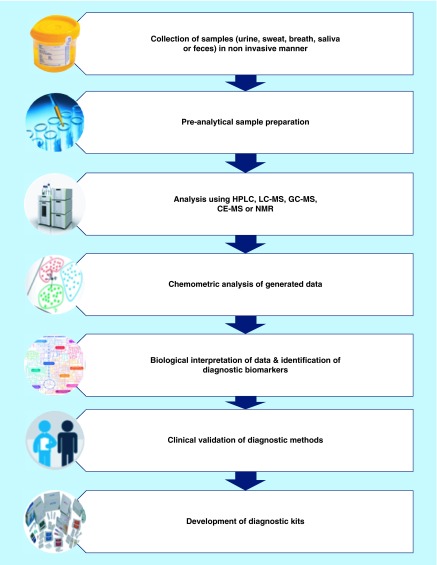
**General workflow for development of painless diagnostic methods using metabolic profiling.**

## Background

The idea of using qualitative metabolite patterns for noninvasive diagnosis of diseases using basic chromatographic techniques dates back to the late 1940s whereby characteristic metabolic patterns in urine and saliva were used for diagnosis [1]. However, the advancement of analytical platforms in the late 1960s ushered the advent of quantitative metabolic profiling. Since its inception the field of metabolic profiling has grown considerably in terms of its applications and contributions to systems biology research. Metabolic profiling is often described by closely related terms such as metabonomics, metabolic phenotyping, metabolomic fingerprinting and metabolomics which are often used interchangeably in contemporary scientific studies [2,3]. Metabolic profiling provides a powerful tool for gaining valuable insight into functional biology, toxicology, pharmacology and hence aids in diagnosis of diseases [4,5]. Metabolic profiling is complementary to genomics and proteomics as it measures the perturbed metabolic end points due to genetic, environmental, pharmacological or pathological influences while in genomics and proteomics, more upstream biological events are typically profiled and studied [6]. It involves the analysis of various biological matrices using suitable analytical platforms. Metabolic profiling can be carried out with a global nontargeted approach as well as with a targeted approach. In targeted metabolic profiling, alterations in the levels of a specific class of metabolites or metabolites belonging to a specific metabolic pathway are ascertained using an appropriate analytical technique [7,8]. In global nontargeted metabolic profiling, metabolites belonging to diverse metabolic pathways are profiled. The metabolites that are determined in nontargeted approach belong to various chemical classes such as organic acids, amino acids, fatty acids, amines, sugars, sugar alcohols, steroids, nucleic acid bases, phospholipids and other miscellaneous substances. So, multiple complementary analytical techniques are often utilized for nontargeted metabolic profiling of biological matrices, in order to cover as much of metabolic space as possible. So, multiple complementary analytical techniques are often utilized for nontargeted metabolic profiling of biological matrices, in order to cover as much of metabolic space as possible [9,10]. Analytical platforms that are commonly used for metabolic profiling include NMR spectroscopy and MS based techniques like direct infusion MS, GC–MS, LC–MS or CE–MS. In addition to these techniques other methods like Fourier transform infrared (FTIR) spectroscopy, LC with ultraviolet or coulometric detection and CE with ultraviolet detection have also been used for metabolic profiling.

Once the biological samples for metabolic profiling are collected they are usually stored at -80°C until further analysis to prevent sample degradation. Selection of sample preparation technique depends upon the type of analytical platform used for metabolic profiling. Although for NMR spectroscopy minimal sample pretreatment is required, the data generated are difficult to process and interpret. In case of LC–MS-based methods, sample cleanup step is required before analysis to eliminate matrix effect. GC–MS methods are not susceptible to matrix effect but lacks throughput because of the extensive sample preparation involved [7].

Global nontargeted metabolic profiling results in the generation of huge and complex datasets containing a large number of variables. Multivariate statistical techniques or chemometric tools are indispensable for the analysis of such datasets. Of the different chemometric methods available such as hierarchical clustering, partitional clustering, artificial neural networks, support vector machine, evolutionary-based algorithms and regression trees, projection-based chemometric methods are extensively used [11].

In spite of the potential applications of metabolic profiling, it has some intrinsic limitations and shortfalls which limit its widespread adaptability in clinical practices. The main lacuna lies in the standardization of collection procedure of biological samples such as urine and feces as various factors like sex, age, life style factors, health status and physical exercise present challenges in analysis and interpretation of acquired data. Moreover selection of proper sample preparation techniques, analytical method development, raw data analysis and integration with other ‘omic’ sciences present various challenges in metabolic profiling [12–16].

Diagnostic methods using invasive and minimally invasive techniques have certain limitations and shortfalls of their own. For instance, endoscopic diagnostic techniques are invasive and may cause iatrogenic injury to patients [17]. Moreover, minimally invasive techniques such as collection of blood or tears, involve trained personnel and the pain associated renders it unpopular among the young population and even among certain adults. So there is an ardent necessity of developing noninvasive diagnostic techniques in order to improve patient compliance and thereby to encourage the active participation of patients for diagnosis.

Blood, urine and tissue are the commonly used biomatrices for metabolic profiling. Biomatrices such as urine, exhaled breath, saliva, sweat and fecal extract can be obtained in a noninvasive manner. Therefore metabolic profiling of these biomatrices holds immense potential and promise for noninvasive diagnosis of diseases [10]. Previously Medina *et al*. [18] authored a review which was focused mainly on the diagnostic metabolomic biomarkers and associated pathways identified only from clinical trials. This review article is an effort to collate and summarize both preclinical and clinical noninvasive metabolic profiling studies carried out so far to develop diagnostic methods of human diseases.

## Urine as a biomatrix for noninvasive metabolic profiling

Urine sample collection is noninvasive by nature and is not restricted by volume. Urine can also be used for measurement of time-resolved, dynamic or temporal data which are an essential attribute for the investigation of the pathogenesis, progression and prognostication of acute and chronic diseases. The metabolite composition of urine is also influenced by other factors such as diet and physiological parameters such as age, gender and demographic characteristics. In some instances, urine may consist of xenobiotics and their metabolites, which can lead to additional complexities in downstream data analysis. Therefore, proper study design in urinary metabolic profiling is of utmost importance to acquire meaningful data and consequent interpretation. Baseline characteristics involved in study groups, sample collection and storage conditions should be carefully controlled and standardized according to the research hypothesis. Diurnal variations in urine caused by diet and lifestyle factors can be nullified by collection of first-pass urine [19].

Urine-based noninvasive metabolic profiling studies have been predominantly used for the diagnosis of different types of cancers such as prostate cancer, lung cancer, liver cancer, bladder cancer, gastric cancer and breast cancer (please refer to Table 1). Urine-based metabolomics in conjunction with other ‘omics’ was used for noninvasive diagnosis of prostate cancer and the method developed outperformed prostate-specific antigen in diagnosis potential [20]. Several studies have indicated that a panel of the urinary nucleosides identified with metabolic profiling performs better with cancer diagnosis than the traditional single tumor biomarker as evident in case of liver cancer [21], breast cancer [22,23] and lung cancer [24,25]. A noninvasive sensitive method to identify volatile organic metabolites as biomarkers that can accurately diagnose the onset of breast cancer was developed by Silva *et al*. [26]. Diagnostic methods of bladder cancer, like cystoscopy and urinary cytology, have a lot of shortfalls. As cystoscopy is expensive and invasive, it lacks patients’ compliance and often fails in detecting recurrent bladder cancer. Urine-based noninvasive metabolic profiling has shown immense potential in replacing the existing conventional diagnostic methods even in distinguishing muscle invasive and nonmuscle invasive bladder cancer [27–30]. Large scale screening and diagnosis for gastric cancer using endoscopy lacks practicality because of the expenses involved, patient compliance and ease of administration. A urinary metabolic profiling approach was found to be useful for the effective diagnosis of gastric cancer and exhibited higher accuracy and sensitivity than carbohydrate antigen 19–9 and carcinoembryonic antigen. Moreover, 4-hydroxyphenylacetate, alanine, phenylacetylglycine, mannitol, glycolate and arginine levels were significantly correlated with cancer T stage and along with hypoxanthine level were able to discriminate healthy from the gastric cancer patients [31,32].

Urine-based noninvasive metabolic profiling studies have also exhibited immense potential in the diagnosis of human metabolic disorders as well as in born errors of metabolism in infants (please refer to Table 2) [33]. Metabolic profiling of infant urine was applied for the diagnosis of organic acidurias and relevant biomarker discovery [34]. Urinary metabolic profiling was also used for screening of 5-day-old newborns for neonatal galactosuria [35]. Nontargeted metabolic profiling by analysis of urine samples collected from infants has been successfully used in the diagnosis of some inborn metabolic disorders such as cystinuria, maple syrup urine disease, adenylosuccinate lyase deficiency and galactosemia [36]. In order to identify urinary biomarkers associated with nutritional rickets in children and to establish a noninvasive diagnosis method, urinary metabolic profiling along with metabolic pathway analysis was used to investigate the metabolic alterations associated with the disease [37]. Wei *et al*. carried out urinary metabolomic profiling for the identification of metabolites relevant for noninvasive diagnosis and stratification of prediabetic stage, which might be imperative in customized treatment for diabetics [38]. Although determination of phenylalanine/tyrosine ratio in blood has been conventionally utilized in diagnosis of phenylketonuria, there are limitations presented by its invasive nature and associated false positives. Recently, a urinary metabolic profiling-based diagnostic method for phenylketonuria has been developed to overcome these limitations [39].

Urine-based metabolic profiling has also been extensively used for noninvasive diagnosis of other diseases and conditions (please refer to Table 3). Gestational hypertensive disorders of pregnancy, especially preeclampsia might contribute to maternal morbidity. A combination of maternal characteristics with the noninvasive urinary metabolic profiling of hippurate/creatinine level significantly improved the prediction rates of preeclampsia [40,41]. A preliminary study by Blydt-Hansen *et al*. indicated that urinary metabolic profiling has the potential to be a sensitive, specific and noninvasive diagnostic method for T-cell-mediated rejection in pediatric kidney transplant recipients. This method was found to be better than the method involving serum creatinine, with minimal confounding parameters introduced by other allograft injury processes [42]. Crohn's disease along with ulcerative colitis makes up the majority of inflammatory bowel diseases (IBD). In the last few years, urinary metabolic profiling studies have been extensively utilized for gaining more insight into metabolic pathways perturbed in IBD which in turn has provided a potential diagnostic and patient stratification tool for IBD [43]. A preliminary study involving urinary metabolic profiling exhibited potential in the diagnosis of hepatitis C virus infection in patients [44]. Similarly a urinary metabolic profiling study exhibited potential for the clinical diagnosis of concomitant depression in hepatitis B virus infected patients. The six relevant biomarkers namely pyruvate, isobutyrate, *N*-methylnicotinamide, alpha-hydroxybutyrate, acetoacetate and malonate might be helpful for developing a validated diagnostic method for the above purpose [45]. Dipstick urinalysis is the conventional semi-quantitative method for diagnosis urinary tract infection but lacks diagnostic accuracy [46]. A recent study by Lam *et al*. involving urine-based targeted metabolic profiling of microbial-mammalian co-metabolite trimethylamine demonstrated its potential for the etiological diagnosis of *Escherichia coli* associated urinary tract infection [47]. Pike *et al*. demonstrated the auxillary role of urinary steroid metabolic profiling in the diagnosis of familial precocious puberty, a subgroup of true precocious puberty [48]. Clinical and preclinical studies involving murine models have already demonstrated the potential of urinary metabolic profiling in improving the diagnosis of rheumatic diseases such as rheumatoid arthritis, spondyloarthritis, systemic lupus erythematosus and osteoarthritis [49]. Urinary metabolic profiling has been successfully utilized in the diagnosis of interstitial cystitis and bacterial cystitis [50].

## Saliva as a biomatrix for noninvasive metabolic profiling

The ease, cost-effectiveness and noninvasive nature of collection, make saliva an ideal bio-matrix for noninvasive metabolic profiling studies. Its functional biology in the oral cavity is well established. Advances in metabolic profiling have resulted in the identification and characterization of an array of salivary metabolites such as amino acids, lipids, antioxidants, polyamines, vitamins and ethylphosphate [51]. These metabolites enter the saliva through extracellular and intracellular routes, carrying real-time information from several organs and thereby provide a potential means of diagnosis of diseases. Saliva is relatively free from confounding factors generally involved in metabolic profiling, such as hemoglobin level, blood pressure, gender and BMI thus making it an ideal bio-matrix for attaining accurate real-time diagnostic results. However, the salivary metabolic profile is influenced by factors such as smoking [52] and diurnal variation [53]. For the development of basic, translational and clinical research in saliva, bioinformatics tools such as ‘Saliva Ontology’, ‘SDxMart’ as well as databases such as ‘Saliva-omics Knowledge Base’ developed by the National Institute of Dental and Craniofacial Research, USA and the National Cancer Institute, USA are available. This has provided efficient data management system and web resources, which in turn has boosted research in salivary metabolic profiling [54]. These factors have contributed to the expansion of the range of salivary metabolic profiling for the diagnosis of both oral and systemic diseases. Salivary metabolic profiling is applicable for diagnosis of local diseases such as oral cancers, Sjogren's syndrome, periodontal diseases as well as distant diseases like autoimmune, metabolic diseases, viral/bacterial infections (please refer to Table 4). Moreover, this provides a means for the development of effective diagnostic devices for clinical application [55–59].

Salivary metabolic profiling has resulted in the identification of unique metabolic signatures of carbohydrate, lipid metabolism and oxidative stress associated with periodontal diseases such as gingivitis and periodontitis, which are associated with Type 2 diabetes mellitus. This in turn could be used for diagnosis and monitoring of both diabetes and periodontal disease [61]. Moreover, other salivary metabolic profiling studies indicated a strong association of Type 2 diabetes with individual salivary biomarkers such as 1,5-anhydroglucitol [63] and d/l-Lactic acid isomer ratio [64]. This could be used as noninvasive diagnostic biomarkers for Type 2 diabetes mellitus.

Salivary metabolic profiling has led to the identification of potential diagnostic biomarkers for Sjogren's syndrome. Sjogren's syndrome is characterized by chronic inflammation and destruction of salivary glands. The study captured the associated perturbations in salivary metabolite pattern, especially a reduction in the level of marker metabolites such as glycine, tyrosine, uric acid, fucose etc. as compared with health controls [65].

Salivary metabolic profiling in conjunction with serum based metabolic profiling was able to provide diagnostic biomarkers of neurodegenerative dementia such as Alzheimer's disease, frontotemporal lobe dementia and Lewy body disease [66].

Early diagnosis of oral squamous cell carcinoma and associated precancerous lesions such as oral lichen planus and oral leukoplakia is essential for increasing the survival rate of patients. Several studies utilizing salivary metabolic profiling have demonstrated the diagnostic potential of several metabolite based biomarkers like propionylcholine, *N*-Acetyl-l-phenylalanine, sphinganine, phytosphingosine, S-carboxymethyl-l-cysteine, γ-aminobutyrate, phenylalanine, valine, n-eicosanoic acid and lactate [67–69]. Apart from oral cancer metabolic profiling of saliva samples has also resulted in the identification of disease-specific markers of pancreatic cancer and breast cancer [60].

## Exhaled breath as a biomatrix for noninvasive metabolic profiling

The fast, accurate and noninvasive diagnosis of respiratory diseases comprises a challenge to the clinicians, and the clinical management can be complicated by the lack of knowledge of the different types of respiratory diseases. Exhaled breath contains a complex mixture of volatile organic compounds (VOCs) in the gas phase which are generated by respiratory tract itself or pulmonary circulation. Thus they could potentially represent metabolite-based diagnostic biomarkers for lung diseases. For instance metabolic profiling of noninvasive VOCs has been used to diagnose lung cancer, infectious diseases, asthma and chronic obstructive lung diseases (please refer to Table 5). Exhaled breath condensate metabolite analysis could be also used as a noninvasive method for the study of pleural fluid. One of the confounding factors associated with the analysis of exhaled breath is the high amount of moisture present in the biomatrix. Moreover the diagnostic accuracy of exhaled breath analysis for some lung diseases like acute respiratory distress syndrome (ARDS) has been validated by preclinical murine models [70]. However, the main limitations to its applicability in regular clinical practice are the standardization of baseline characteristics in study populations as well as establishment of standard collection and analytical procedures [71–79]. Collection of exhaled breath usually utilizes electronic nose such as gold nanoparticles and sensors-based electronic nose. Breath samples are usually stored in different containers with Tedlar bag being the most common [77].

Basanta, *et al*. demonstrated that it was possible to distinguish patients with and without chronic obstructive pulmonary disease from healthy controls using noninvasive metabolic profiling of exhaled breath [72]. A need for diagnostic biomarkers of the ARDS necessitated metabolic profiling study of exhaled breath of patients. The study was able to identify three breath metabolites namely octane, acetaldehyde and 3-methylheptane which could be used for diagnosis of ARDS [76].

Metabolic profiling study of exhaled breath condensate in children was applied successfully to differentiate asthma phenotypes, with a particular focus on severe inflammatory state. Metabolites related to retinoic acid, adenosine and vitamin were found to be significant for separation of different asthma subtypes. In a similar study, a panel of eight metabolite based biomarkers, namely, 1-(methylsulfanyl)-propane, ethylbenzene, 1,4-dichlorobenzene, 4-isopropenyl-1-methylcyclohexene, 2-octenal, octadecyne, 1-isopropyl-3-methylbenzene and 1,7-dimethylnaphtalene were found to be relevant for effective separation of asthmatic and healthy children [80–84].

Early diagnosis of lung cancer is desirable for reduction of high rate of morbidity associated with the disease. Metabolic profiling of exhaled breath VOCs could provide a noninvasive method for widespread screening for early diagnosis of lung cancer. *In vitro* cell culture under hypoxic conditions, have been used for gaining knowledge and identification of biomarkers relevant to lung cancer diagnosis [90]. Clinical extrapolation of metabolic profiling studies of exhaled breath VOCs [79] have lead to the identification of diagnostic biomarkers of lung cancer such as n-Dodecane [91], 1-butanol, 3-hydroxy-2-butanone (for nonsmall cell lung cancer) [92], hexadecanal [93], 5-(2-methyl-)-propyl-nonane, 2,6-di-tert-butyl-4-methyl-phenol, 2,6,11-trimethyl-dodecane; 8-hexyl-hexadecanal; pentadecane [94]. Similar diagnostic applicability of metabolic profiling of exhaled breath can be found in case of pleural mesothelioma [95].

Metabolic profiling of exhaled breath has shown noninvasive diagnostic potential in other forms of cancer such as breast cancer and liver cancer. The diagnostic potential of exhaled breath metabolites in breast cancer have been demonstrated by some studies. These have resulted in the identification of significant diagnostic biomarkers such as 3-methylhexane, dec-1-ene, caryophyllene, naphthalene, trichloroethene [85], hexanal, heptanal, octanal and nonanal [86]. Metabolic profiling of exhaled breath has also been used for noninvasive diagnosis of liver cancer and has resulted in the identification of diagnostic biomarkers such as 3-Hydroxy-2-butanone, hepatocellular carcinoma and decane [89].

Metabolic profiling of breath VOCs has been utilized to separate breath metabolite patterns of patients with and without lower respiratory tract infections thus exhibiting its diagnostic potential in respiratory tract infections [97].

Metabolic profiling of exhaled breath condensate was used to discriminate cystic fibrosis (CF) from healthy controls thus exhibiting its diagnostic potential [87]. CF-related diabetes (CFRD) may result from CF and it is characterized by rapid decline in lung condition. Moreover, targeted metabolic profiling of glucose in exhaled breath condensate has the potential of successfully diagnosing CF and CFRD [88]. NMR-based metabolic profiling studies have been utilized for differentiating primary ciliary dyskinesia from CF [96] as well as patients suffering from pulmonary Langerhans cell histiocytosis from healthy subjects [62].

## Feces as a biomatrix for noninvasive metabolic profiling

Fecal samples consist of diverse range of endogenous metabolites, gut microbiota metabolites with different physicochemical properties and volatility. Metabolic profiling of feces not only helps in the discovery of disease biomarkers but also in providing an insight about the relationship of the gut microbiome and human health. The transient stay of feces in the colon and rectum makes it an ideal biomatrix for gaining information about the health and pathological state of colon and rectum. Metabolic profiling of feces has the potential for noninvasive diagnosis of IBD and colorectal cancer (please refer to Table 6). However, the complex nature of feces present challenges in sample collection, sample storage, sample preparation and development of analytical methods [98–100].

Metabolic profiling of fecal volatile organic metabolites helped in providing information about the etiology of the disease and also resulted in the identification of diagnostic biomarkers of irritable bowel syndrome [101]. Crohn's disease along with ulcerative colitis makes up the majority of IBD. Discrimination of Crohn's disease from ulcerative colitis as well differentiation of IBD from irritable bowel syndrome is imperative for patient stratification and effective clinical management. However, conventional diagnostic methods lack accuracy in this matter. Metabolic profiling studies of feces have already shown diagnostic potential in case of the above-mentioned bowel conditions [101,102].

Colorectal cancer is one of the major forms of cancer in terms of morbidity in developed countries. The application of metabolic profiling to fecal water extracts has shown potential as a diagnostic tool for detecting colorectal cancer. NMR-based metabolic profiling of fecal water extracts from patients with colorectal cancer and healthy individuals, was able to identify potential diagnostic markers such as short-chain fatty acids (acetate and butyrate) and amino acids (proline and cysteine) [103]. A similar study using lyophilized feces resulted in the identification of relevant fecal biomarkers of colorectal cancer [104]. Metabolic profiling of fecal samples using GC–MS also exhibited diagnostic potential in the detection of colorectal cancer [105].

The db/db mouse model, consisting of a nonfunctional leptin receptor, is a commonly used murine model of Type 2 diabetes mellitus. Nontargeted metabolic profiling of fecal extracts of db/db and wild-type mice revealed a wide range of metabolites related to diabetes such as sulfate containing fatty acids (cyprinol sulfate), sulfate containing bile acids (sulfocholic acid, oxocholic acid sulfate, taurocholic acid sulfate) and steroidal metabolites. These metabolites were able to discriminative wild-type from db/db mice. Furthermore, including *N*-acyl taurines were altered in diabetic mice, enabling us to focus on S-containing metabolites, especially the sulfate and taurine conjugates of bile and fatty acids [106].

## Unconventional biomatrices for noninvasive metabolic profiling

Metabolic profiling of hair samples collected from three different rodent model namely hypertensive model rats (SHR/Izm), stroke-prone SHR (SHRSP/Izm) and Wistar Kyoto (WKY/Izm) was carried out using LC–MS. The results obtained were subjected to chemometric analysis which in turn resulted in successful group separation. This study demonstrated the future potential of hair as a feasible biomatrix for noninvasive metabolic profiling for diagnostic purpose [107].

Sweat is an unconventional biomatrix when it comes to biomarker discovery and diagnosis of diseases. So far sweat electrolyte concentration has been successfully used for the diagnosis of CF [108]. Recently, metabolic profiling of sweat has been carried out for noninvasive diagnosis of lung cancer. This resulted in the identification of significant marker metabolites such as a trisaccharide phosphate, suberic acid, a tetrahexose, a trihexose, nonanedioic acid and monoglyceride (22:2) [109].

## Conclusion

Noninvasive diagnostic methods are essential for clinical management of human diseases and disorders from the point of view of patient compliance and comfort. A general workflow for the development of painless diagnostic methods using metabolic profiling has been shown in Figure 1. The information provided in this review affirmed the established as well as the potential applications of metabolic profiling in the noninvasive diagnosis of human diseases and disorders. The most commonly used biomatrices that are collected and used for this purpose are urine, exhaled breath, saliva and fecal extract [10]. Apart from these popular biomatrices, there exist some unconventional biomatrices such as sweat [109] and hair [107] that can be used for noninvasive diagnostics. The application of metabolic profiling for noninvasive diagnostics can be observed in different forms of cancer, metabolic disorders like diabetes, in born errors of metabolism, infectious diseases, neurodegenerative disorders rheumatic diseases and pulmonary diseases. Preclinical studies and small scale clinical studies have provided evidence of the diagnostic potential of such metabolic profiling studies. However, further large scale clinical studies are essential for validation of some diagnostic methods and to extend its application in regular clinical practice. Therefore, metabolic profiling of biomatrices which can be collected noninvasively holds the key for painless diagnosis of diseases and disorders.

## Future perspective

The diagnostic potential of metabolic profiling of biomatrices which can be collected painlessly in certain diseases and disorders has been asserted strongly by several preclinical and small scale clinical studies. The predictive power of the noninvasive diagnostic methods depends upon study design, baseline characteristics and confounding factors which make such comparison and meta-analysis difficult to establish. So, clinical studies are required in future for validation of the potential diagnostic methods. For the implementation of metabolic profiling as a nonnvasive diagnostic tool in clinical practices large scale prospective clinical studies are required in distant future. The main thrust areas involve diseases such as IBD [43], hepatitis C virus infection [44], depression associated with hepatitis B virus-infected patients [45], neurodegenerative disorders [66], ARDS [76], pleural mesothelioma [95], respiratory tract infections [97], nonalcoholic fatty liver disease [110,111] and rheumatic diseases [49].

As metabotype varies from one biological sample to another as well as between different studies, validation of multivariate statistical model for a particular disease or disorder is of utmost importance. Validation of the already available results especially in the field of metabolic profiling of saliva, exhaled breath and urine could lead to the development of high-throughput noninvasive point-of-care and bedside diagnostic devices. Development of such devices are feasible in some forms of cancer like breast cancer, lung cancer, oral cancer and pancreatic cancer [77,105] and gestational hypertensive disorders especially preeclampsia [40,41].

Future research in the field of metabolic profiling using unconventional biomatrices such as sweat and hair could increase the popularity of these biomatrices in terms of their diagnostic applications [107,108]. The tear film covering the epithelial cells of the ocular surface is essential for visual function and its composition is a good indicator of the surface-health of the eye. However, the clinical method of tear collection is minimally invasive and results in confounding factors in metabolic profiling. All these have restricted its feasibility as a biomatrix for noninvasive metabolic profiling. However, innovation in the collection method of tear samples could minimize the associated discomfort and could extend its utility for noninvasive diagnosis of eye diseases [112,113]. In the future, research in sensor technologies could also help in the advancement of point-of-care diagnostic devices especially in the field of exhaled breath metabolic profiling. Thus noninvasive metabolic profiling has paved the way for an exciting field of future research in diagnostic methods and devices.

**Table 1.** **Urinary metabolic profiling studies used for noninvasive diagnosis of different types of cancer.**

**Type of cancer**	**Analytical platform used**	**Ref.**
Bladder cancer	LC–MS, GC–MS	[26–28]
Breast cancer	HPLC, LC–MS, GC–MS	[22,23]
Gastric cancer	GC–MS, NMR	[31,32]
Liver cancer	NMR, MS, LC–MS, GC–MS	[21]
Lung cancer	NMR, MS, LC–MS, GC–MS	[24,25]
Prostate cancer	LC–MS	[20]

**Table 2.** **Urinary metabolic profiling studies used for noninvasive diagnosis of metabolic disorders and in born errors of metabolism.**

**Diseases or disorders**	**Analytical platform used**	**Ref.**
Cystinuria, maple syrup urine disease, adenylosuccinate lyase deficiency, galactosemia	LC–MS	[3]
Neonatal galactosuria	GC–MS	[35]
Nutritional rickets	LC–MS	[37]
Organic acidurias	GC–MS	[34]
Phenylketonuria	GC–MS	[39]
Prediabetic subtypes	GC–MS	[38]

**Table 3.** **Urinary metabolic profiling studies used for noninvasive diagnosis of miscellaneous diseases and disorders.**

**Diseases or disorders**	**Analytical platform used**	**Ref.**
Depression associated with hepatitis B virus infection	NMR	[45]
Familial precocious puberty	GC–MS	[48]
Gestational hypertensive disorder especially preeclampsia	NMR	[41]
Hepatitis C virus infection	NMR	[44]
Immuno-rejection in kidney transplantation	MS	[42]
Inflammatory bowel diseases	NMR, MS, LC–MS, GC–MS	[43]
Interstitial and bacterial cystitis	NMR, MS	[50]
Rheumatoid arthritis, spondyloarthritis, systemic lupus erythematosus, osteoarthritis	NMR, MS	[49]
Urinary tract infection	NMR	[46,47]

**Table 4.** **Salivary metabolic profiling studies used for noninvasive diagnosis of different diseases and disorders.**

**Diseases or disorders**	**Analytical platform used**	**Ref.**
Breast cancer	CE–MS	[60]
Neurodegenerative dementias (Alzheimer's disease, frontotemporal lobe dementia, Lewy body disease)	CE–MS	[57]
Pancreatic cancer	CE–MS	[60]
Periodontal diseases like (gingivitis and periodontitis) in diabetic patients	GC–MS, LC–MS	[53]
Primary Sjogren's syndrome	GC–MS, LC–MS	[55,56]
Squamous cell carcinoma and related precancerous lesions like oral lichen planus and oral leukoplakia	LC–MS, CE–MS	[58–59,61–62]
Type 2 diabetes mellitus	NMR, MS, LC–MS, GC–MS	[54]

**Table 5.** **Metabolic profiling studies using exhaled breath for noninvasive diagnosis of different diseases and disorders.**

**Diseases or disorders**	**Analytical platform used**	**Ref.**
Acute respiratory distress syndrome	GC–MS	[68]
Asthma	NMR	[80–84]
Breast cancer	GC–MS	[85,86]
Chronic obstructive pulmonary disease	GC–DMS, NMR	[71,72]
Cystic fibrosis and related diabetes	IMS–TOF-MS, NMR	[87,88]
Hepatocellular carcinoma	GC–MS	[89]
Lung cancer	GC–MS	[79,90–94]
Pleural mesothelioma	GC–MS	[95]
Primary ciliary dyskinesia	NMR	[96]
Pulmonary Langerhans cell histiocytosis	NMR	[62]

GC–DMS: Gas-chromatography–differential mobility spectrometry; IMS–TOF-MS: Ion-mobility–time of flight-mass spectrometry.

**Table 6.** **Metabolic profiling studies using feces for noninvasive diagnosis of different diseases and disorders.**

**Diseases or disorders**	**Analytical platform used**	**Ref.**
Irritable bowel syndrome and Inflammatory bowel diseases	GC–MS, NMR	[101,102]
Colorectal cancer	GC–MS, NMR	[103–105]
Type-2 diabetes mellitus	FT–ICR–MS, LC–MS	[106]

FT–ICR–MS: Fourier transform–Ion cyclotron resonance–Mass spectrometer.

**Executive summary**

**Background**

Painful diagnostic methods using invasive or minimally invasive techniques may cause patient discomfort and iatrogenic injury to patients.
There is a need for development of noninvasive diagnostic methods in order to improve patient compliance and patient participation.
Metabolic profiling of biomatrices like urine, exhaled breath, saliva, sweat and feces which can be collected in a painless manner could be used for noninvasive diagnosis of diseases and disorders.

**Urine for noninvasive metabolic profiling**

Urinary metabolic profiling studies have been predominantly used for the diagnosis of different forms of cancers, metabolic disorders and in born errors of metabolism.
Urinary metabolic profiling has also been used for noninvasive diagnosis of gestational preeclampsia, infectious diseases and rheumatic diseases.

**Saliva for noninvasive metabolic profiling**

Salivary metabolic profiling is applicable for diagnosis of local diseases like oral cancers, Sjogren's syndrome and periodontal diseases.
The applicability of salivary metabolic profiling could also be extended for the diagnosis autoimmune disorders, metabolic diseases and infectious diseases.

**Exhaled breath for noninvasive metabolic profiling**

Exhaled breath consists of a complex mixture of volatile organic compounds in the gas phase and could act as diagnostic biomarkers for diseases.
Metabolic profiling of exhaled breath has been used to diagnose lung cancer, liver cancer, chronic obstructive lung diseases, cystic fibrosis and respiratory tract infections.

**Feces for noninvasive metabolic profiling**

Feces provide information about the interaction of the gut microbiome and human health.
The transient stay of feces in colon makes it an ideal biomatrix for gaining information about its health and pathological state.
Metabolic profiling of feces has the potential for the diagnosis of irritable bowel syndrome, inflammatory bowel diseases and colorectal cancer.

**Unconventional biomatrices for noninvasive metabolic profiling**

Metabolic profiling of unconventional biomatrices such as hair and sweat holds future potential for noninvasive diagnosis of diseases and disorders.

**Conclusion**

The most commonly used biomatrices include urine, exhaled breath, saliva and feces in addition to less popular biomatrices like sweat and hair.
The application of metabolic profiling for noninvasive diagnostics can be observed in cancer, metabolic disorders, in born errors of metabolism, infectious diseases, neurodegenerative disorders, rheumatic diseases and pulmonary diseases.
Future large scale clinical studies are necessary for validation of some diagnostic methods and to extend its application in regular clinical practice.
